# Sources of vitamin D and calcium in the diets of preschool children in the UK and the theoretical effect of food fortification

**DOI:** 10.1111/jhn.12277

**Published:** 2014-10-03

**Authors:** V. L. Cribb, K. Northstone, D. Hopkins, P. M. Emmett

**Affiliations:** ^1^ Centre for Child and Adolescent HealthSchool for Social and Community MedicineUniversity of BristolBristolUK; ^2^ School for Social and Community MedicineUniversity of BristolBristolUK; ^3^ Nutrition and Dietetic DepartmentSouthampton General HospitalHampshireUK

**Keywords:** Avon longitudinal study of parents and children, calcium, dietary intakes, food fortification, preschool children, vitamin D

## Abstract

**Background:**

Dietary intakes of vitamin D are very low in the UK. Dietary calcium is also necessary to promote bone health. The fortification of foods with vitamin D could be a safe and effective way of increasing intake.

**Methods:**

Diets of preschool children, 755 at 18 months and 3.5 years, from the Avon Longitudinal Study of Parents and Children were assessed using dietary records completed by parents. Energy, vitamin D and calcium intakes were calculated. Multinomial logistic regression was used to estimate the odds ratio for being in the highest/lowest quartile of intake. Intakes were recalculated to test different fortification regimes.

**Results:**

Vitamin D intakes were low; all children were below the UK and US dietary recommendations. Calcium intakes decreased between the two ages as a result of reduced milk consumption. Children in the lowest quartile for vitamin D intake at 18 months were twice as likely to remain in that quartile at 3.5 years (odds ratio = 2.35; 95% confidence interval = 1.56–3.55). The majority of foods provide no vitamin D with fat spreads and milk as the main sources. The contribution from breakfast cereals increased, from 6% to 12%, as a result of the increased consumption of fortified cereals. Dairy foods provided the highest contribution to calcium at 18 months but were less important at 3.5 years. Theoretical intakes from different fortification regimens suggest that milk fortified at 2 μg 100 g^–1^ vitamin D would provide most children with adequate but not excessive intakes.

**Conclusions:**

Dietary vitamin D intakes were very low and calcium intakes were mostly adequate. Fortification of milk with vitamin D could be a good way to boost intakes.

## Introduction

Vitamin D status has become an increasingly controversial issue in recent years (Pearce & Cheetham, 2010; Clarke & Page, 2012) with a sharp rise in the UK and USA of the bone disease rickets, which was once considered to have been eradicated (Wagner & Greer, 2008; Davies & Shaw, 2011). Rickets is a disease of bone development in childhood resulting in soft bones that bow under the weight of the body (Wharton & Bishop, 2003). It is primarily caused by a deficiency in vitamin D and/or dietary calcium (Bishop, 1999). Vitamin D is required by the body to regulate the absorption of calcium and phosphorus from the diet and their deposition in bone. There is an expanding list of other health claims for vitamin D, which are covered in the ESPGHAN position paper on vitamin D in relation to paediatric populations (Braegger *et al*., 2013). The results of studies suggest that these other health claims may be an over interpretation of the results of observational surveys, assigning causality inappropriately (Harvey & Cooper, 2012) and the British Paediatric and Bone group has published a position statement to try to clarify its position on vitamin D deficiency, which they say should relate only to the effect of vitamin D on the skeleton (Arundel *et al*., 2012).

Vitamin D is often known as the ‘sunshine’ vitamin because exposure of the skin to sunlight leads to its synthesis, with approximately 80% of the recommended intake supplied to the body in this way (Webb *et al*., 2010). Vitamin D is a fat soluble vitamin and it occurs in two forms; D_2_ and D_3_. Vitamin D_2_ (ergocalciferol) comes from food and D_3_ (cholecalciferol) is produced in the skin following sun exposure (Papandreou *et al*., 2009). Both forms are hydroxylated in the liver to 25‐hydroxyvitamin D (25‐OH‐D) and serum levels are used as a measure of vitamin D status. If sunlight exposure is limited, there is increased dependence on dietary sources to provide adequate intakes (Webb *et al*., 2010), especially in vulnerable groups, during winter months (Glerup, 2000; Webb *et al*., 2010). However, there are only a few foods that are naturally rich in vitamin D; good sources include liver and fatty fish such as salmon, mackerel and sardines, whereas other foods such as red meat and eggs provide marginal amounts (O'Connor & Benelam, 2011). Therefore, foods fortified with vitamin D are likely to be major contributors to dietary intake; in the UK, these include margarine and some breakfast cereals (Foundation, 2011). Fortification policies for vitamin D in food vary worldwide (Calvo *et al*., 2004); in the UK, fortification of margarine and infant formula is mandatory, although some manufacturers voluntarily fortify other products. Some other countries fortify milk successfully (Calvo *et al*., 2004), although policies appear to be inconsistent (Tylavsky *et al*., 2006). Furthermore, fortification is a contentious issue. The OPTIFORD (Optimal Strategy for Vitamin D Fortification) European project was set up to examine whether vitamin D fortification is a feasible strategy and to determine the optimum level of fortification needed for prevention of insufficiency without reaching upper tolerable levels, particularly in the under 5‐year‐olds (Tylavsky *et al*., 2006). It concluded that substantially more research is required before a consensus can be reached.

An alternative view is that vitamin D supplements should be given to those at risk of deficiency (Wagner & Greer, 2008). The UK Department of Health recommends that all children up to the age of 5 years should receive vitamin D supplements (Department for Health, 2009), although the supplements themselves are not always available and the advice may not be followed through by health professionals; consequently, uptake is poor. In a survey carried out for a charity in 2012, only 49% of health professionals who were questioned knew about the recommendations (Harvey & Cooper, 2012). In the UK National Diet and Nutrition Survey (NDNS) of 2008/11, only 12% of children aged 1.5–3 years were taking supplements of vitamin D (Bates *et al*., 2012). Other countries also recommend that children receive vitamin D supplements, although this advice varies (Calvo *et al*., 2004; Tylavsky *et al*., 2006). Again, uptake of supplements is low; in the German DONALD study, only 7% of 2–3‐year‐olds were taking them (Sichert‐Hellert *et al*., 2006). Although the main source of vitamin D should be sunlight exposure, there is legitimate concern about excessive exposure in relation to skin cancer with the resultant use of high factor sunscreen and/or covering up limiting the amount of vitamin D synthesised. This has been highlighted by a study among 187 children who presented at an orthopaedic clinic in the UK, 32% of whom had low circulating vitamin D levels (Davies *et al*., 2011).

The aim of the present study is to assess the sources of vitamin D and calcium in the diets of UK preschool children, in comparison to energy intake from core and noncore foods, using a longitudinal birth cohort. The secondary aim is to assess whether possible fortification levels for vitamin D are likely to be safe and effective for preschool children.

## Materials and methods

### Subjects

Subjects were participating in the Avon Longitudinal Study of Parents and Children (ALSPAC), an on‐going UK longitudinal birth cohort study designed to investigate the health and development of children. ALSPAC has been described fully elsewhere (http://www.bristol.ac.uk/alspac Golding *et al*., 2001; Boyd *et al*., 2013). Briefly, pregnant women were eligible to participate if they had an expected delivery date between April 1991 and December 1992 and were resident in the former Avon Health Authority in South West England. A cohort of 14 541 pregnancies, resulting in 13 988 children alive at 12 months, was established and mothers comprising a 10% random sample from the final 6 months of recruitment were invited to bring their infants to a research clinic for in‐depth assessments during their early years. These children have provided the data for this study. Of the 1453 children who ever attended this part of ALSPAC, 755 (52%) have provided dietary data at both 18 months and 3.5 years. The mothers of these children were biased toward having high education levels (A‐levels or above) compared to mothers of the whole ALSPAC cohort (44.3% and 35.4%, respectively). Ethical approval for the study was obtained from the ALSPAC Law and Ethics Committee and the Local Research Ethic Committees.

### Dietary assessment

Dietary data were collected from the child's main carer at two clinic visits; when the child was aged 18 months in 1994 and when aged 3.5 years in 1996. The full methods have been described elsewhere (Cowin & Emmett, 2000; Emmett *et al*., 2002). In summary, prior to the clinic visit, carers were asked to record in a structured diary (using household measures) all food and drink consumed by the child over 3 days; 2 weekdays and 1 weekend day (self‐selected and not necessarily consecutive), with a description of any leftovers. At the clinic, they were interviewed by a nutrition fieldworker to clarify any anomalies. At 3.5 years a short questionnaire was also included asking about the use of vitamin supplements, types of fat spread normally used on bread and other details of foods commonly eaten.

The completed diaries were coded by a nutritionist using the computer software dido (Diet In, Data Out) originally developed by the MRC Human Nutrition Research Unit (Price *et al*., 1995). The portion sizes used were informed by the descriptions in the food records with information from the second edition of the ‘Food Portion Sizes’ book (Ministry of Agriculture & Fisheries & Food, 1993) and from given weights of manufactured foods. The databank used for nutrient analysis included the 5th edition of McCance and Widdowson's food tables (Holland *et al*., 1991a,b) and supplements (Holland *et al*., 1988, 1989, 1991a,b, 1992a,b, 1993; Chan *et al*., 1994, 1995). Additional up‐to‐date nutrient information was obtained from the NDNS database (Gregory *et al*., 1995), as well as manufacturers' information, and covered all foods eaten. The coded diaries were checked against the originals by a different nutritionist. The average energy, vitamin D and calcium content of the foods and the food groups that the child ate over 3 days, and the percentage contribution to these nutrients from each food group were calculated. Nutrient intakes from vitamin supplements were not included in this analysis. The calculated nutrient intakes were very similar to those from weighed food records collected from NDNS, a nationally representative sample of similarly‐aged children (Gregory *et al*., 1995; Cowin & Emmett, 2000; Emmett *et al*., 2002). NDNS was updated in 2008–2011 and shows similar nutrient intakes to the previous study (Bates *et al*., 2012).

### Statistical analysis

To make comparisons between foods eaten at the two different ages possible, nutrient contents of food groups were divided individually by the respective average total energy intake (MJ day^–1^). The Means (SD) was calculated for energy‐adjusted nutrients from each food group at each age. Foods were divided into core and noncore foods, as defined by the Australian Guide to Healthy Eating (Koh *et al*., 2010). Core foods are nutrient‐rich and include: (i) bread, cereals, rice, pasta and noodles; (ii) vegetables, legumes and potatoes (not chips); (iii) fruit; (iv) yoghurt, cheese and milk; and (v) meat, fish, poultry, eggs and nuts. Noncore foods (listed below) tend to be energy‐dense and nutrient‐poor and include all processed foods with added sugar or fat (e.g. biscuits, cakes, chips, coated fish and processed meats). Fat spreads are also classified as noncore despite being a major source of fat soluble vitamins. Water and milk are classified as core beverages, and all other drinks are classified as noncore (Bell *et al*., 2005). Comparisons between the age groups were made in the 755 children with complete data, using paired *t*‐tests. Vitamin D and calcium intakes at both time‐points were categorised into quartiles. The tracking of both nutrients from 18 months to 3.5 years was assessed using a multinomial logistic regression to estimate the odds ratio for being in the highest/lowest quartile for the nutrient at both ages. All statistical analyses were performed using spss, version 18 (SPSS Inc., Chicago, IL, USA).

### Fortification of foods with vitamin D

Theoretical intakes of vitamin D were calculated using fortification levels for vitamin D in various commonly eaten foods based on the OPTIFORD project (Tylavsky *et al*., 2006). Vitamin D intakes for each child were increased by the theoretical fortification applied to each food in the amount consumed. The levels of intake likely in these children after fortification were compared to the UK Reference Nutrient Intake (RNI) (Department of Health, 1991) and the US Recommended Dietary Allowance (RDA) (US Departments of Agriculture & Health & Human Services, 2010) and European (European Commission – Health & Consumer Protection Directorate General, 2002) and US upper tolerable limits. The upper limit is the highest level of daily consumption of vitamin D that current evidence has shown to cause no risk of adverse health effects; in the USA, the upper level intake is set at 62.5 μg day^−1^ in 1–3‐year‐olds (O'Connor & Benelam, 2011; Ross *et al*., 2011), whereas, in Europe, this is 25 μg day^−1^ in 0–2‐year‐olds (European Commission ‐ Health & Consumer Protection Directorate General, 2002). Our data were compared with both limits to assess whether any children are likely to reach toxic levels of intake as a result of fortification.

## Results

### Sample characteristics

A total of 1026 children provided food records at 18 months and 863 at 3.5 years, with 755 children having them at both ages. As part of the diary, parents indicated whether the children were taking any vitamin supplements; 18% of children consumed vitamin supplements at 18 months, falling to 11% at 3.5 years.

### Dietary intakes

Total vitamin D intakes were low but increased slightly from 18 months to 3.5 years; however, calcium intakes decreased between the two ages (Table 1). This was the result of a reduction in contribution from milk (providing 108 mg Ca MJ^−1^ at 18 months; 69 mg MJ^−1^ at 3.5 years). All children in the present study were below the UK and US recommendations for vitamin D intake at both ages (Department of Health, 1991; US Departments of Agriculture & Health & Human Services, 2010). Only a very small percentage of children did not meet the UK calcium requirements. However, US calcium recommendations are set at a much higher level and 33% were below this at 18 months, rising to 45% at 3.5 years. Children in the lowest quartile for vitamin D intake at 18 months were more than twice as likely to remain in that quartile at 3.5 years [odds ratio (OR) = 2.35; 95% confidence interval (CI) = 1.56–3.55]. There was less evidence of tracking of high intakes 1.40 (95% CI = 0.95–2.14). For calcium, tracking was more likely; children in the lowest quartile at 18 months were almost three times as likely to continue in that quartile [OR = 2.82; 95% CI = 1.90–4.17). This was similar for the highest quartiles (OR = 2.47; 95% CI = 1.68–3.65).

**Table 1:** Energy, vitamin D and calcium intakes [mean (SD)] from parentally‐completed dietary records at 18 months and 3.5 years and the proportion of children falling below target intakes

	18 months (*n *= 1026)	3.5 years (*n *= 863)
Mean energy (MJ)	4.62 (0.94)	5.67 (1.08)
Mean vitamin D (μg)	1.56 (1.57)	1.79 (1.36)
Mean calcium (mg)	806 (256)	768 (285)
Current UK Recommendations^a^		
RNI^b^ for vitamin D (μg day^−1^)	7	7
% below RNI recommendation	98	100
Mean intake as % RNI	22	26
UK LRNI^c^ for calcium (mg day^−1^)	200	200
UK EAR^d^ for calcium (mg day^−1^)	275	275
UK RNI for calcium (mg day^−1^)	350	350
% below RNI recommendation	4	4
% LRNI for calcium	0	0
Mean intake as % RNI	230	219
Current US recommendations^e^		
RDA^f^ for vitamin D (mg day^−1^)	15	15
% below recommendation	100	100
RDA for calcium (mg day^−1^)	700	700
% below RDA recommendation	33	45

a Department of Health (1991).

b RNI (Reference Nutrient Intake) is the amount of a nutrient that is enough to ensure the needs of almost all the population (97.5%) are being met.

c LNRI (Lower Reference Nutrient Intake) is the amount of a nutrient that is enough for only the small number of people who have a low requirement (2.5%).

d EAR (Estimated Average Requirement) is an estimate of the average requirement for a nutrient.

e US Department of Agriculture and US Department of Health and Human Services (2010).

f RDA (Recommended Dietary Allowance) is the average daily dietary intake level that is sufficient to meet the requirements of almost all (approximately 98%) healthy individuals.

### Vitamin D and calcium intakes from core and noncore foods at each age

Energy‐adjusted vitamin D and calcium intakes from core and noncore foods together with the percentage contribution from food sources for energy and both nutrients are shown in Tables 2 and 3. At 18 months, core foods provided 36% of the vitamin D intake, rising to 41% at 3.5 years. Noncore foods made a substantial contribution to this very low overall intake and this increased between the two ages (33–48%). For calcium, core foods provided 85% and 81% at the two ages and the contribution from noncore foods increased from 11% to 17%. Formula milk and baby foods have not been presented in Tables 2 and 3; at 18 months, 19% of vitamin D and 3% of calcium came from these foods; they were not consumed at 3.5 years.

**Table 2:** Energy‐adjusted vitamin D [mean (SD)], calcium [mean (SD)] and percentage contribution to intake and to energy intake from core foods consumed by children (*n *= 755) at both 18 months and 3.5 years (food group intakes compared between the two ages by a paired *t*‐test)

Core foods	18 months					3.5 years				
	Contribution to energy (%)	Vitamin D (μg MJ^−1^)	%a	Calcium (mg MJ^−1^)	%b	Contribution to energy (%)	Vitamin D (μg MJ^−1^)	%a	Calcium (mg MJ^−1^)	%b
Bread, cereals, rice and pasta										
Group total	15.3	0.026 (0.04)^d^	5.9	10.9 (6.7)^c^	6.3	17.9	0.047 (0.04)^d^	12.5	13.8 (7.9)^c^	10.1
Vegetables, potatoes (no fat) and legumes										
Group total	4.0	0.008 (0.02)	2.9	3.4 (3.5)^c^	1.9	3.4	0.008 (0.02)	3.1	2.8 (3.5)^c^	2.1
Fruit, fruit juice										
Group total	5.7	0.00 (0.00)	0.0	2.7 (3.1)	1.5	5.1	0.00 (0.00)	0.0	2.5 (2.6)	1.8
Yoghurt, cheese and milk										
Group total	31.8	0.035 (0.02)^d^	8.8	128.1 (56.8)^c^	73.4	21.7	0.023 (0.02)^d^	6.3	88.2 (45.2)^c^	65.3
Meat, fish, poultry and eggs										
Group total	5.8	0.059 (0.08)	17.6	2.7 (4.1)	1.5	5.6	0.065 (1.0)	18.8	2.7 (4.7)	2.0
Core foods total	62.6	0.13 (0.09)	35.2	147.7 (54.7)^c^	84.6	53.7	0.14 (0.11)	40.7	109.9 (44.5)^c^	81.3

%a Percentage contribution to vitamin D and %b percentage contribution to calcium.

Significant difference (*P* < 0.001) in energy adjusted nutrient intakes between 18 months and 3.5 years when same lowercase letter (c, d) is used.

**Table 3:** Energy‐adjusted vitamin D [mean (SD)], calcium [mean (SD)] and percentage contribution to intake from noncore foods consumed by children (*n *= 755) at both 18‐months and 3.5 years (food group intakes compared between the two ages by a paired *t*‐test)

Noncore foods	18 months (*n *= 1026)					3.5 years (*n *= 863)				
	Contribution of energy (%)	Vitamin D (μg MJ^−1^)	%a	Calcium (mg MJ^−1^)	%b	Contribution of energy (%)	Vitamin D (μg MJ^−1^)	%a	Calcium (mg MJ^−1^)	%b
Puddings, cakes, pastries, biscuits, ice cream, confectionery and savoury snacks										
Group total	17.8	0.027 (0.04)^d^	5.9	13.6 (9.0)^c^	7.5	24.7	0.038 (0.06)^d^	9.4	16.4 (8.9)^c^	12.0
Processed meat, fish and poultry										
Group total	4.4	0.022 (0.03)^d^	5.9	2.8 (2.9)^c^	1.7	5.9	0.029 (0.03)^d^	6.3	3.7 (3.8)^c^	2.7
Potatoes with fat, baked beans										
Group total	4.3	0.00 (0.00)	0.0	2.1 (2.8)	1.3	5.5	0.00 (0.00)	0.0	2.1 (2.4)	1.6
Fat spreads, soup and sauces										
Group total	4.5	0.073 (0.07)^d^	20.6	0.9 (2.1)	0.6	5.5	0.093 (0.07)^d^	28.1	0.7 (1.8)	0.7
Noncore foods total	31.0	0.12 (0.08)^d^	32.4	19.5 (9.9)^c^	11.1	41.6	0.16 (0.09)^d^	43.8	23.0 (9.5)^c^	17.0

%a Percentage contribution to vitamin D and %b percentage contribution to calcium.

Significant difference (*P* < 0.001) in energy adjusted nutrient intakes between 18 months and 3.5 years when same lowercase letter (c, d) is used.

Intakes of vitamin D from both core and noncore foods were low, with most foods providing none. Fat spreads and milk were the main sources at each time‐point. Interestingly, the percentage contribution to vitamin D from breakfast cereals increased considerably between the ages. This was explained by a change in the types of breakfast cereals consumed; at 18 months, the majority of children consumed plain cereals that were not fortified with vitamin D: at 3.5 years, they were more likely to consume the types of cereal that had been fortified (usually by 2.1 μg 100 g^–1^) (Holland *et al*., 1991a,b).

The core foods, milk, yoghurt and cheese provided the highest contribution to calcium at 18 months. The contribution from bread was also high; this is because, in the UK, bread is fortified with calcium during production. At 3.5 years, calcium intakes from milk decreased and cheese increased. However, there was an overall drop in calcium intake from the dairy group (*P *< 0.001). From noncore food items, puddings and confectionery were the main sources of calcium at each age.

### The contribution of particular foods

Full‐fat polyunsaturated margarine (7.9 μg 100 g^–1^) contributed the most vitamin D, followed by full‐fat nonpolyunsaturated margarine. Butter provided very little vitamin D at either time‐point because it contains only 0.76 μg 100 g^–1^. Whole milk provided the majority of the vitamin D from the milk group (0.03 μg 100 g^–1^). Children who had been switched from whole milk to semi‐skimmed received much less (0.01 μg 100 g^–1^).

Whole milk provided the most calcium at both ages (450 mg at 18 months; 300 mg at 3.5 years) compared to semi‐skimmed (36 mg at 18 months, rising to 91 mg at 3.5 years). The contribution from bread rose between 18 months and 3.5 years, with white bread contributing the most (21.5 mg at 18 months, 37.7 mg at 3.5 years).

### Fortification of food items

Theoretical dietary intakes using different fortification levels of vitamin D in several foods are shown in Table 4 with ranges of intake and comparisons with RNI and tolerable upper limits in Table 5. If milk were fortified at a level of 5 μg 100 g^–1^, this would increase the intake of vitamin D from milk in these children from 0.14 to 21.7 μg day^−1^ at 18 months; slightly less at 3.5 years. Fortifying fat spreads to 10 μg 100 g^–1^ would double intakes from this source at both ages. Using the full fortification of all the foods would vastly increase the vitamin D intake but would lead to the possibility of toxic intakes compared to the upper tolerable limit suggested in Europe. Our data suggest that the safest level of fortification of milk, in addition to the other fortified foods, would be at a level of 2 μg 100 g^–1^ where very few children had theoretical intakes below the RNI or above the European upper tolerable level.

**Table 4:** Estimated intake of vitamin D from milk, fat spreads, bread and breakfast cereals in diets of children at 18 months and 3.5 years with and without fortification of that food

	18 months		3.5 years	
	Intakes before (vitamin D μg)	Intakes after fortification (vitamin D μg)	Intakes before (vitamin D μg)	Intakes after fortification (vitamin D μg)
Milk – fortified to 2 μg 100 g^–1^	0.14 (0.11)	8.56 (4.59)	0.10 (0.13)	6.86 (4.46)
Milk – fortified to 5 μg 100 g^–1^	0.14 (0.11)	21.7 (11.3)	0.10 (0.13)	17.2 (11.1)
Fat spreads – fortified to 10 μg 100 g^–1^ a	0.34 (0.32)	0.60 (0.43)	0.53 (0.44)	0.93 (0.61)
Yoghurt – fortified to 5 μg 100 g^–1^	0.01 (0.01	2.02 (2.08)	0.01 (0.02)	2.0 (2.2)
Bread – fortified to 2 μg 100 g^–1^	0.00 (0.00)	0.67 (0.44)	0.00 (0.00)	1.06 (0.58)
Breakfast cereals (all) to 2 μg 100 g^–1^ b	0.11 (0.16)	0.37 (0.35)	0.23 (0.24)	0.47 (0.41)
Total vitamin D from food sources including all food fortified above and milk fortified at 5 μg 100 g^–1^	1.53 (1.51)	25.99 (11.0)	1.72 (0.82)	22.42 (11.4)

a Fat spreads currently fortified at 5 μg 100 g^–1^.

b Some breakfast cereals currently fortified.

**Table 5:** Estimated total dietary intakes of vitamin D at 18 months and 3.5 years at different levels of fortification of milk, compared to adequate intakes and upper tolerable limits of intake

	18 months (*n *= 1026)				3.5 years (*n *= 863)			
	Range	% below RNI^a^	Number over US upper tolerable limit of 62.5 μg	Number over European upper tolerable limit of 25 μg	Range	% below RNI^a^	Number over US upper tolerable limit of 62.5 μg	Number over European upper tolerable limit of 25 μg
Before fortification	0.09–15.59	98	0	0	0.12–23.02	99	0	0
Foods fortified as in above table except milk	0.42–17.86	83	0	0	0.27–17.78	76	0	0
Foods fortified as in above table – milk at 1.5 μg 100 g^–1^	1.21–24.04	13	0	0	1.13–31.17	20	0	4
Foods fortified as in above table – milk at 2 μg 100 g^–1^	1.21–31.44	9	0	13	1.13–37.45	15	0	15
Foods fortified as in above table – milk at 5 μg 100 g^–1^	1.21–76.15	4	3	563	1.13–78.89	5	7	301

a RNI (Reference Nutrient Intake) is the amount of a nutrient that is sufficient to ensure the needs of almost all the population (97.5%) are being met.

## Discussion

This longitudinal study identified very low intakes of vitamin D from diet and declining intakes of calcium between age 18 months and 3.5 years in UK children. There was evidence of tracking of dietary intake from one age to the next. Despite the recommendation (Department of Health, 1991) that children should receive vitamin D supplements, very few were taking them. There may be implications for these continued low intakes on optimal health in later childhood and adulthood, particularly when there is inadequate sunlight exposure. We found that, in theory, the fortification of milk with vitamin D at 2 μg 100 g^–1^ would improve the diets of preschool children to levels that were adequate but not excessive.

The low dietary vitamin D intakes found in the present study (22–26% of the recommended intake) are consistent with the results from the 2008/11 NDNS study of childhood diet in UK, which shows in cross‐section that vitamin D intakes are extremely low; with young children obtaining only 26% of the intake recommended to originate from the diet (Department of Health, 1991) and average intake being increased only marginally by supplement use, which was only at a level of 12% in this population (Bates *et al*., 2012). Calcium intake in NDNS 2009/11 was 223% of the RNI again similar to the preset study. Intakes in other European countries were also comparable; a study of 696 Belgian preschoolers, using diet records, found adequate calcium intakes but very low vitamin D intakes (Huybrechts *et al*., 2011) and, in 900 2–3‐year‐olds collected from 1986 to 2003 in the German DONALD study, even in consumers of supplements (7%), only half the recommended intake of vitamin D was achieved (Sichert‐Hellert *et al*., 2006). Furthermore, studies worldwide have shown that many people, both children and adults, have inadequate serum levels of vitamin D (Holick, 2006); typically, 36% of otherwise healthy young adults had inadequate serum vitamin D levels (<50 nmol L^−1^). These other studies have not had longitudinal data and so the present study is unique in showing that intakes of both vitamin D and calcium track as children get older.

There are very few foods that are naturally rich in vitamin D and this was reflected in the sources of vitamin D in the diets of these children. Milk, meat, fish and eggs were the major core food providers, with fortified fat spreads being the major noncore source. Certain types of breakfast cereal have long been fortified in UK and these added to intakes in the older children. Some manufacturers are currently increasing fortification of breakfast cereals to 1.25 μg 100 g^–1^ (or 25% of UK RDA) but mainly for types that contain added flavourings and sugar and are aimed at children. This is welcome but it would be more beneficial to fortify all breakfast cereals. Calcium is much more abundant in foods, with core dairy foods being particularly important sources. However, these children consumed less calcium at 3.5 years than they had at 18 months. At the same time, the amount of energy consumed from noncore foods that provided only 11–17% of the calcium rose from 31% to 42%, with the diet becoming more energy and less nutrient‐dense.

It is surprising that, in the 20 years subsequent to the study data being collected, there are no differences in levels of intake of either vitamin D or calcium and no increase in uptake of vitamin D supplements in the UK despite the continuous official recommendations. Perhaps the very few food sources for vitamin D hinder the possible increase of intake via food and perhaps parents are wary about giving supplements to their children or are not receiving advice to do so. In this respect, fortification of foods provides a potential solution to improve intakes of vitamin D (O'Connor & Benelam, 2011). Commonly fortified foods, which vary with country, include milk, margarine, breakfast cereals and bread (O'Mahony *et al*., 2011). In the USA and Canada, where milk is voluntarily fortified, there is evidence that its consumption improves the vitamin D status of adults but only by modest amounts, with the taking of supplements being much more effective (Calvo & Whiting, 2003). The present study of very young children provides evidence of the potential increases in vitamin D intakes, if food fortification were to become more widespread in the UK. The fortification of milk, in particular, would be the most effective way of enabling young children, who are eating the types of diet recorded in the present study, to achieve adequate intake levels. However, the likelihood of reaching the upper tolerable limit for vitamin D intake must also be considered and our data suggest that fortifying milk at 2 μg 100 g^–1^ would be reasonably safe.

Some studies have directly investigated the effect of vitamin D fortification: one a partially blinded, randomised trial examined the effect of either red meat or fortified toddler milk intake on vitamin D status in 181 New Zealand infants (aged 12–20 months) (Houghton *et al*., 2011). After the 20‐week trial, there were higher serum concentrations for 25‐OH‐D in the milk groups compared to the meat group, and it was concluded that an intake of 4 μg day^−1^, as provided by the fortified milk, was sufficient to ensure that children had adequate intakes of vitamin D (Houghton *et al*., 2011). A study in Finnish adults, aiming to develop a model for optimal food fortification, suggested that it would be difficult to find an appropriate fortification level for the whole population if only selected foods were fortified. The alternative would be to fortify all fortifiable foods with 1.2–1.5 μg vitamin D 100 kcal^−1^ 419kJ^−1^, although this would result in intakes being above the upper tolerable limit in some people (Hirvonen *et al*., 2007).

A strategy aimed at increasing uptake of vitamin D supplements is the currently recommended alternative, although it is not very effective at present (an uptake of approximately 12% in the most recent survey; Bates *et al*., 2012). To increase this level would require a major education campaign for health professionals and parents and it would need the guaranteed availability of supplements as either being provided free or at an affordable price. A further problem is that supplements tend to be taken by advantaged rather than disadvantaged groups, thus increasing inequality (Gregory *et al*., 1995).

The main strengths of the present study are its relatively large sample size and the comprehensive dietary data available at two time‐points during early childhood. However, the study was conducted in one geographical area of the UK and it is possible that the results may not be applicable throughout the country, although the cohort was reasonably representative of the UK population at recruitment (http://www.bristol.ac.uk/alspac) and the dietary data have been shown to be comparable with nationally collected cross‐sectional data in similarly‐aged children (Cowin & Emmett, 2000; Emmett *et al*., 2002). Our data were collected in 1994 and 1996 and it is possible that the diets of children have changed; however, in a repeat of the NDNS in 2009/11, the diets of 1.5–3‐year‐old children had similar average energy content, compared to those assessed previously, and a very similar vitamin D and calcium content to those in the present study, and so it is likely that our findings remain applicable (Bates *et al*., 2012).

## Conclusions

The present study confirms that dietary vitamin D intakes are very low in young children and that calcium intake falls as children move from the toddler stage to preschool. This longitudinal change has not been reported prevously and was mainly the result of the increased intake of nutrient‐poor noncore foods. Unless a great deal more effort is put towards increasing the very low uptake of vitamin D supplements, the most effective way of improving dietary vitamin D status in UK children would appear to be via food fortification.

**Conflict of interests, source of funding and authorship**

The authors declare that they have no conflicts of interest.

The UK Medical Research Council (Grant ref: 74882), the Wellcome Trust (Grant ref: 092731) and the University of Bristol provide core support for ALSPAC. This research was specifically funded by Wyeth Nutrition, although it was carried out independently. VC, KN, DH and PE have occasionally received research funding and PE has received consultancy funding from Pfizer Nutrition Ltd, Plum Baby and Danone Baby Nutrition (Nutricia Ltd).

PE designed the data collection tools, monitored the data collection, designed the analysis and contributed to interpretation and revision. PE serves as guarantor. VC performed the analyses, and also drafted and revised the manuscript. KN advised on the statistical methods and contributed to interpretation and revision. DH contributed to the interpretation by providing expertise from clinical practice. All authors critically reviewed the manuscript and approved the final version submitted for publication.
